# CyTRANSFINDER: a Cytoscape 3.3 plugin for three-component (TF, gene, miRNA) signal transduction pathway construction

**DOI:** 10.1186/s12859-016-0964-2

**Published:** 2016-04-08

**Authors:** Gianfranco Politano, Francesca Orso, Monica Raimo, Alfredo Benso, Alessandro Savino, Daniela Taverna, Stefano Di Carlo

**Affiliations:** Department of Control and Computer Engineering, Politecnico di Torino, Corso Duca degli Abruzzi 24, Torino, 10129 Italy; Molecular Biotechnology Center (MBC), Via Nizza, 52, Torino, 10126 Italy; Dept. Molecular Biotechnology and Health Sciences, University of Torino, Via Nizza, 52, Torino, 10126 Italy; Center for Complex Systems in Molecular Biology and Medicine, Via Accademia Albertina, 13, Torino, 10123 Italy

**Keywords:** microRNA, Signal transduction pathways, Data fusion, Cytoscape, Network analysis, Pathway analysis, Network modules

## Abstract

**Background:**

Biological research increasingly relies on network models to study complex phenomena. Signal Transduction Pathways are molecular circuits that model how cells receive, process, and respond to information from the environment providing snapshots of the overall cell dynamics. Most of the attempts to reconstruct signal transduction pathways are limited to single regulator networks including only genes/proteins. However, networks involving a single type of regulator and neglecting transcriptional and post-transcriptional regulations mediated by transcription factors and microRNAs, respectively, may not fully reveal the complex regulatory mechanisms of a cell. We observed a lack of computational instruments supporting explorative analysis on this type of three-component signal transduction pathways.

**Results:**

We have developed CyTRANSFINDER, a new Cytoscape plugin able to infer three-component signal transduction pathways based on user defined regulatory patterns and including miRNAs, TFs and genes. Since CyTRANSFINDER has been designed to support exploratory analysis, it does not rely on expression data. To show the potential of the plugin we have applied it in a study of two miRNAs that are particularly relevant in human melanoma progression, *miR-146a* and *miR-214*.

**Conclusions:**

CyTRANSFINDER supports the reconstruction of small signal transduction pathways among groups of genes. Results obtained from its use in a real case study have been analyzed and validated through both literature data and preliminary wet-lab experiments, showing the potential of this tool when performing exploratory analysis.

**Electronic supplementary material:**

The online version of this article (doi:10.1186/s12859-016-0964-2) contains supplementary material, which is available to authorized users.

## Background

Network representation of intracellular biological systems, considering molecular components within a cell as nodes (e.g., genes, proteins, miRNA, etc.) and their direct or indirect interactions as links, is steadily gaining interest because of its potential to represent, characterize, and model a wide range of intricate natural systems and phenomena.

Among the different types of biological network models proposed in the literature [1], we are interested in *Signal Transduction Pathways* (STP) [2, 3]. A cell is highly responsive to specific chemicals in its environment. Broadly, signal transduction pathways can be viewed as molecular circuits. They model how cells receive, process, and respond to information from the environment toward a biological identified end result, thus providing snapshots of the (overall) cell dynamics. The number of these processes shows how many ways the organism can react and respond to its environment. Therefore, discovering new STPs is an important task to contribute to the current knowledge of the cell behavior.

The traditional approach to identify molecular components of a signaling network is through gene knockout experiments and epistasis analysis [4]. In such experiments, an organism is engineered to suppress the expression of one or more genes in order to study the resulting perturbation in the cell dynamics. Although these experiments are effective to identify simple direct signaling activities, more complex signaling circuitries are difficult to identify and understand. Moreover this analysis is time-consuming, expensive, and sometimes the results can be misinterpreted [5].

Computational approaches for modeling and reconstruction of STPs are currently a hot research area. STPs have been modeled through modular kinetic simulations of biochemical networks [6], and detailed integration of biochemical properties of the pathways [7]. Bayesian networks applied to multi-variate expression data have also been used to infer signaling pathways [8]. More recently, PPI networks have been largely used to reconstruct signaling transduction pathways [9–13]. In general these methods try to extract STPs from PPI networks, which are known to be affected by a high rate of false-positive and false-negative interactions. The use of expression data is used to mitigate this uncertainty.

Most of the attempts to reconstruct STPs focus on gene/protein based networks. However, networks involving a single type of regulator may not fully reveal the complex regulatory mechanisms of a cell. Complexity strongly increases when STPs include post-transcriptional regulation mediated by microRNAs (miRNAs) interacting with different transcription factors (TFs). It is predicted that miRNAs regulate approximately 30 % of the human protein-coding genome [14], they are therefore highly important in modeling the cell regulation. Only a few attempts to reconstruct STPs including miRNAs, TFs, and mRNAs can be found in the literature [15, 16].

Motivated by this, we have developed CyTRANSFINDER, a new Cytoscape 3.3 [17] plugin able to construct three-component signal transduction pathways with the presence of miRNAs, TFs and genes starting from public available regulatory information. Rather than trying to construct big networks as proposed in other studies, CyTRANSFINDER focuses on reconstruction of small signal transduction pathways based on user defined regulatory patterns. These pathways may be of direct use to drive exploratory analysis enabling to better understand experimental data and to further drive laboratory experiments. Formally the problem addressed by CyTRANSFINDER is the following: *“Given two set of genes, to discover a set of STPs connecting each gene of the first set with each gene of the second set according to a signaling pattern set by the user.”* Recurring signaling patterns have been widely studied in gene regulatory networks as well as other real-world complex systems scenarios [18], because of their central role in driving regulatory responses by specific functions [2]. This assumption is based on the expectation that designs with higher modularity have higher adaptability and therefore higher survival rates [19], thus suggesting that modularity can spontaneously arise under changing environments [20], which eventually results in extremely complex systems made of simple basic building blocks [19].

Since CyTRANSFINDER has been designed to support exploratory analysis, it does not rely on expression data. It includes a data-fusion engine that scrapes information from seven online repositories and integrates them to infer candidate pathways. Different filters can be applied to restrict or enlarge the set of produced results based on the specific use cases. The integration with Cytoscape 3.3 features an intuitive user interface that automates complex tasks and makes the plugin a potential software instrument for biologists with limited skills in computer programming and network analysis. Moreover, it enables to further process and analyze the identified networks with the huge ecosystems of network analysis plugins and functions already available in Cytoscape 3.3.

To the best of our knowledge no other Cytoscape plugin offers the functionalities provided by CyTRANSFINDER. A Cytoscape 2.6 plugin implementing a front-end to BIANA (Biologic Interactions and Network Analysis) is the only tool that somehow offers functionalities related to CyTRANSFINDER [21]. BIANA is a general Python framework aiming at integrating information from several external data-sets in network representations that can be visualized through the Cytoscape plugin. However, differently from CyTRANSFINDER, most of the effort given in BIANA is put on the possibility of describing external data sources and rules to integrate data from different sources. It is therefore a more generic software that does not specifically focuses on the problem of reconstructing STPs, as done instead by CyTRANSFINDER. BIANA standalone application appears discontinued from 2013, while the latest plugin update is dated 2009 and the plugin is only compatible with Cytoscape 2.6, which is becoming obsolete.

To show the capability of the plugin, we have applied it to a study of two miRNAs that are particularly relevant in human melanoma progression, *miR-146a* and *miR-214*. Results obtained from CyTRANSFINDER have been analyzed and validated through both literature data and preliminary wet-lab experiments, showing the capability of this tool when performing exploratory analysis.

## Implementation

**Fig. 1:**
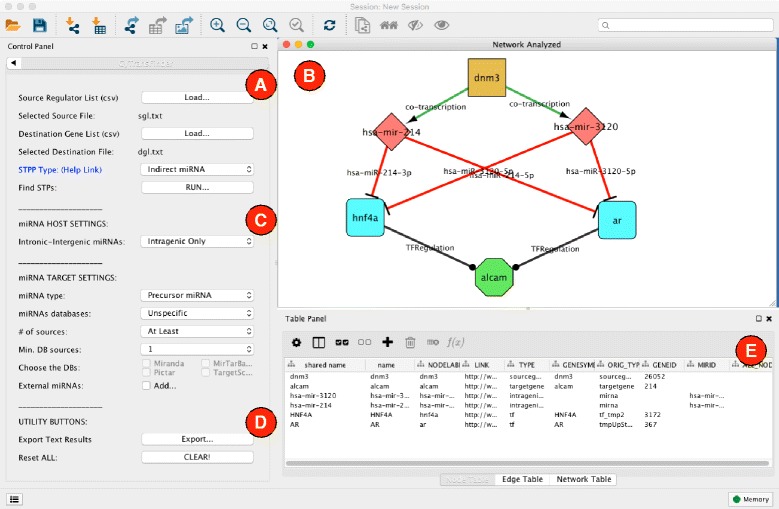
CyTRANSFINDER overview. **a** The main plugin control panel. It allows the user to set the source and destination list of genes and the specific pattern of regulators to search. **b** Shows the graphical output of the plugin that consists of a network connecting source genes with destination genes. Nodes of this network represent genes, TFs and miRNAs. **c** This panel allows to define a set of parameters related to the integration of miRNAs into the generated STPs. They can be used to control the size of the generated networks. **d** This panel allows the user to export the results in the form of a text file including all identified circuits or to delete the current experiment and start with a new one. **e** The Cytoscape node and edge tables. They can be used to access detailed information on the nodes and arcs of the identified STPs

**Fig. 2:**
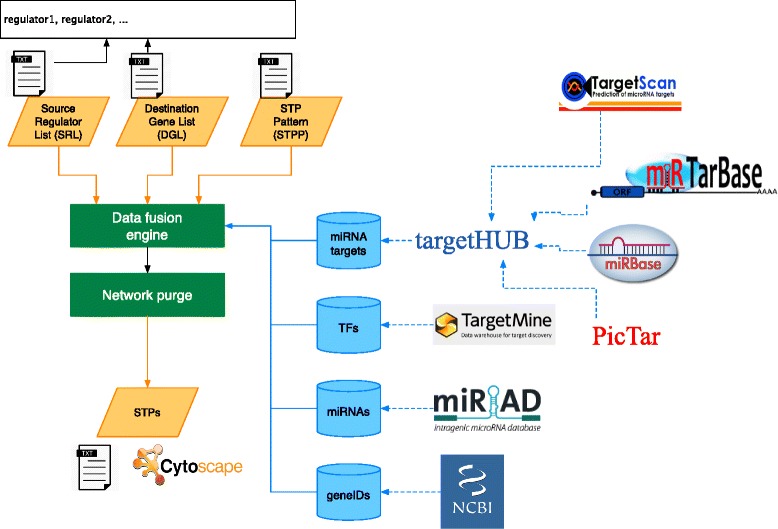
CyTRANSFINDER software architecture. CyTRANSFINDER processes three inputs: (1) the source list of genes (SRL), (2) the destination list of genes (DGL), and (3) the STP pattern (STPP) to be reconstructed. Its fusion engine connects to several on-line repositories to collect regulatory information used to infer STPs connecting source and destination genes according to the selected STP pattern. The identified STPs are then purged to remove duplicated nodes and arcs and the result is returned to the user as a Cytoscape network or exported in the form of a tab separated text file

In its basic setup, CyTRANSFINDER implements STP discovery among two sets of genes into Cytoscape integrating regulatory information on the *Homo sapiens* (human) species. It is developed to work with Cytoscape 3.3. All examples proposed in this paper have been tested with the latest Cytoscape version (Cytoscape 3.3). Once installed from the Cytoscape App Manager, CyTRANSFINDER is available from the Apps menu of Cytoscape. Figure 1 shows a screenshot of CyTRANSFINDER running on a small example whereas Fig. 2 shows the conceptual architecture of the software highlighting its main data sources and computational modules.

CyTRANSFINDER processes three main inputs:

1. *Source Regulator List (SRL)*: is a list of regulators working as root nodes of the inferred STPs. Regulators can be either genes or miRNAs (*DNM3* – Dynamin 3 – NCBI ID: 26052 in the example of Fig. 1).
2. *Destination Gene List (DGL)*: is a list of genes working as leaves of the inferred STPs (*ALCAM* – activated leukocyte cell adhesion molecule – NCBI ID: 214 in the example of Fig. 1).
3. *STP Pattern (STPP)*: is a pattern provided through an internal STPP specification language. It describes a general template of regulators to be identified to connect a source node *sn*∈*SRL* to a destination gene *dn*∈*DGL*.

The user can enter the desired inputs through panel (A) of Fig. 1. Both (SRL) and DGL are provided in a text file formatted as described in Fig. 2. Each gene can be defined by either the *gene symbol* or the *NCBI gene ID*, while miRNAs are defined using the miRBase identifier (e.g., hsa-mir-214). The STPP can be chosen from a list of default patterns or customized as described in the following sections.

RUN button in panel (A) of Fig. 1 starts the STP search. CyTRANSFINDER fusion engine connects to several on-line repositories to collect regulatory information used to infer STPs connecting source and destination nodes according to the selected STPP. The identified STPs are then purged to remove duplicated nodes and arcs and displayed in the form of a network (panel (B) – Fig. 1).

### STPP specification language

A STPP is a general sequence of regulators connecting two genes (or a miRNA to a gene) that are of interest for a given biological question. CyTRANSFINDER provides the user with a simple STPP Specification Language to specify the desired signaling pattern. Three regulators are available in CyTRANSFINDER: (1) gene, (2) TF, and (3) miRNA. The basic regulators are combined into a set of 6 possible interactions that are reported in Table 1 together with the notation used for their specification. They represent realistic biological interactions among the three regulators. The user is free to combine all the interactions of Table 1 in order to describe the desired signaling pattern, which is provided to CyTRANSFINDER in the form of a single text file loaded when setting STPP Type to “Custom STPP” in panel (A) of Fig. 1.

**Table 1:** STPP Specification Language

#	Regulation type	Description	Notation
1	TF ↦ gene	A transcription factor controlling the rate of transcription of a gene	tf,gene
2	TF ↦ TF	A transcription factor controlling the rate of transcription of a another transcription factor	tf,tf
3	TF ↦ miRNA	A transcription factor hosting a miRNA	tf,mirna
4	gene ↦ miRNA	A gene hosting a miRNA	gene,mirna
5	miRNA ↦ gene	A miRNA post-transcriptionally targeting a gene	mirna,gene
6	miRNA ↦ TF	A miRNA post-transcriptionally targeting a TF	mirna,tf

The list of interactions that can be used in CyTRANSFINDER to build a STPP. For each interaction the related notation is reported. The user is free to combine the interactions in order to describe the desired pattern. The first element of the list must be a gene or miRNA and must be preceded by the term “source”, while the last element must a gene and has to be be preceded by the term “target”

**Fig. 3:**
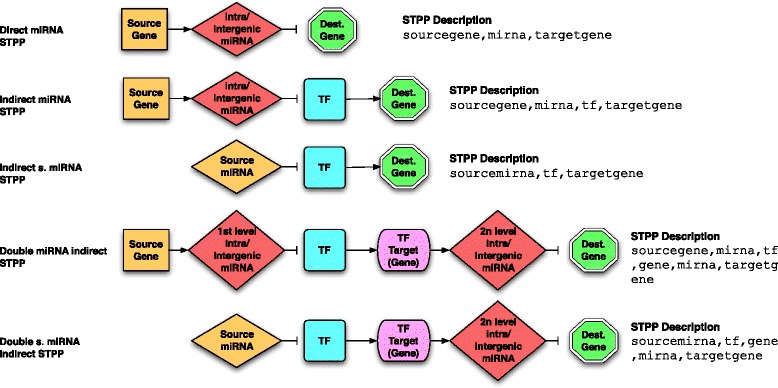
CyTRANSFINDER built-in STPPs. The figure presents the five default STPPs embedded in the plugin. i) *Direct miRNA STPP* is the simplest pattern: a source gene hosts a intragenic mirna miRNA or is located close to the region of an intergenic miRNA, which targets one of the destination genes. ii) *Indirect miRNA STPP*, is pretty similar to the Direct miRNA STPP, but it involves a TF as miRNA mediator for the regulation of the destination genes. iii) the miRNA sourced version of (ii). iv) *Double miRNA* indirect STPP is the most complex pattern. It involves two levels of regulation; the first indirect regulation is modeled on top of an Indirect miRNA STPP, which regulates a Direct miRNA STPP that targets the destination genes. v) The miRNA sourced version of (iv)

Five default STPPs representing common recurring patterns often analyzed in the literature are directly embedded in the plugin (Fig. 3); three STPPs starting from genes and two starting from miRNAs. The *Direct miRNA STPP* is the simplest pattern. A source gene hosts a miRNA which also targets one of the destination genes. The *Indirect miRNA STPP*, is similar to the Direct miRNA STPP, but it involves a TF as miRNA mediator for the destination genes regulation. The *Double miRNA indirect STPP* is the most complex pattern that combines the two previous ones into two levels of indirect regulation: the first one is an Indirect miRNA STPP, which regulates a Direct miRNA STPP that targets the destination gene. Additionally, a version of the *Indirect miRNA STPP* and of the *Double miRNA indirect STPP* starting from a miRNA instead of a gene are available and named *Indirect s. miRNA* and *Double s. miRNA* indirect, respectively.

### Data fusion engine

The RUN button available in panel (A) of Fig. 1 starts the CyTRANSFINDER data fusion engine. The engine first parses the SRL, the DGL and the STPP provided by the user. It then connects to several external repositories to obtain interaction data to search for the existence of the STPP among the genes contained in SRL and DGL.

**Fig. 4:**
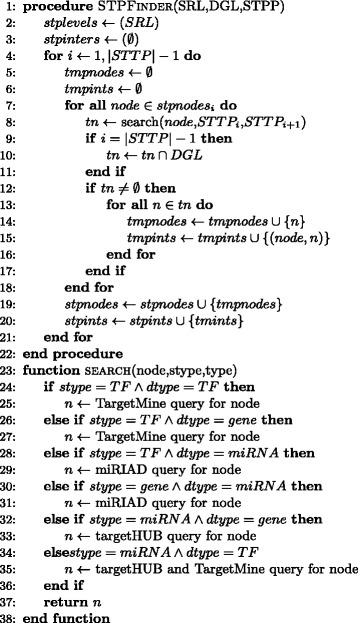
CyTRANSFINDER data fusion algorithm. A pseudocode description of the main steps carried out by the plugin to integrate different data sources and to construct the final STP network

Figure 4 provides a high-level pseudo-code of the implemented data-fusion algorithm. The main algorithm is described in the *STPFinder* procedure (Fig. 4 - lines 1–22). This procedure receives as parameters the source and destination node lists (i.e., SRL and DGL) and the STPP. The STPP is an ordered list of regulators *STPP*=(*r*_1_,*r*_2_,·,*r*_*n*_), with *r*_*i*_∈{*TF,gene,miRNA*}. The produced STPs are organized into a set of levels (*stplevels* in Fig. 4). Each level contains a set of nodes and corresponds to one of the elements of STPP. At the beginning of the search the first level is initialized with the nodes contained in SRL (Fig. 4 - lines 2). Nodes of adjacent levels are connected through a set of interactions (*stpinters* in Fig. 4).

The search procedure is an iterative process that analyzes couples of consecutive STPP elements, i.e., *STPP*_*i*_ and *STPP*_*i*+1_ with *i*∈[1,|*STPP*|−1] (Fig. 4 - lines 4–21). For each node available at level *i* (Fig. 4 - lines 7–18) the procedure searches a set of target nodes to add to level *i*+1 through the search function (Fig. 4 - lines 8). The way this search works (Fig. 4 - lines 23–38) depends on the type of regulators to search at level *i* and *i*+1 of STPP, which in turn requires to connect to different repositories to obtain interaction data. Additional details regarding this process will be provided later in this section. Each target node identified with this procedure is added to the set of nodes at level *i*+1 (Fig. 4 - line 14) and the set of interactions between couples of nodes at the two levels is recorded as well (Fig. 4 - line 15). When reaching the last couple of STPP elements, all identified interactions are finally filtered to limit them to those ending to one of the nodes available in DGL (Fig. 4 - lines 9–11).

At the end of this iterative process, *stplevels* and *stpinters* are used to build a Cytoscape network representing the inferred STPs. *stplevels* contains the set of nodes of the final network organized in levels and *stpinters* contains the set of arcs connecting the different nodes. Panel (B) of Fig. 1 shows an example of network identified when searching for the Indirect miRNA STPP between *DNM3* and *ALCAM*. This network represents the main output provided by CyTRANSFINDER. Different symbols and colors have been used to make it easy to identify the different types of regulators in the network.

The same information can also be exported into a tab separated plaintext file that enumerates all identified STPs (Export button of panel (D) – Fig. 1). Each row of the file reports a single STP (i.e., a signaling chain from one node in SRL to a node in DGL according to the STPP) and each column represents a regulator in the signaling chain (i.e., a gene, miRNA or TF). This file is obtained by searching all possible paths that connect nodes at the source level with nodes at the destination level. This format is particularly helpful for fast data inspection, especially when the number of discovered STPs is high, which eventually results in a very complex network difficult to visualize.

The remaining of this section focuses on the way interactions are obtained and integrated from public repositories.

#### Transcription factors interaction data

Transcription Factors (TFs) related to gene entities are extracted from TargetMine [22] using its RESTful interface, which allows to search for TFs given a target gene. This information is required to search for interactions of type 1,2 and 6 of Table 1. It is important to highlight that TargetMine does not provide any information regarding the up- or down- regulatory activity of a TF; users must eventually resort to manual validation in order to understand the exact regulatory effect.

#### miRNA interaction data

Two different repositories are exploited to retrieve miRNA based interactions.

Interactions of type 3 and 4 from Table 1 are obtained through the miRIAD repository [23]. miRIAD is a web search tool designed to access integrated information concerning intragenic microRNAs and their host genes. The miRIAD database references annotated genes from human genome (hg19) and miRNAs annotated from miRBase (version 19). Given a gene, CyTRANSFINDER uses miRIAD to search for miRNAs “hosted” by the gene. Two types miRNA interactions can be identified with this procedure. The main class is represented by the intragenic miRNAs, which are mapped to intragenic loci of protein coding genes (namely “host genes”). Previous studies have suggested that these miRNAs are transcribed in parallel with their host transcripts [24, 25] therefore creating a direct signaling link. In addition to this, miRIAD enables to search for intergenic miRNAs located in an intergenic region close the the analyzed gene. The relationship between a gene and the intergenic miRNAs is weaker than the one of intragenic miRNAs. Nevertheless, it may represent a valuable information when performing explorative analysis. By acting on the miRNA host type control (panel (C) – Fig. 1) the user is free to work with intragenic only interactions or both intragenic and intergenic interactions.

Interactions of type 5 and 6 from Table 1 are instead obtained from TargetHUB [26]. This web-service provides a programmer friendly interface to access multiple repositories of miRNA target genes with a uniform set of APIs. TargetHUB RESTful interface allows users to interrogate information from four different databases: miRTarBase [27], TargetScan [28], PicTar [29], and miRanda [30]. Using TargetHUB, the list of target genes of a miRNA can be easily retrieved.

CyTRANSFINDER exploits TargetHUB functionalities to allow users to filter miRNA targets (miRNA target settings of panel (C) – Fig. 1). Filtering miRNA targets is a very crucial step during STP discovery. In fact, the lack of miRNA specificity and the large amount of possible miRNA targets (in the order of thousands) may easily increase the complexity of the networks generated by the plugin. A wise usage of filters can dramatically reduce the analysis time. Available filters include the possibility of selecting specific miRNA target databases or to perform majority voting across multiple databases to have a mandatory minimum or exact set of confirmations for the target selection. Moreover, the user can decide to work with regulatory information regarding mature o precursor miRNAs.

Finally, miRNA target settings of panel (C) include an additional control that acts in a opposite way with respect to the other controls, and tries to enlarge the obtained network. This control acts after the full STP search is concluded adding to the network all external miRNAs targeting at least one of the nodes identified in the generated network (i.e., not hosted by one of the network nodes). This option is particularly useful whenever users are focusing on the role of miRNAs in the studied phenomena.

## Results and discussion

In this section we show the capability of CyTRANSFINDER by presenting its application in the framework of a research activity on human melanoma performed by the authors of this paper. In previous studies we and others identified that *miR-146a* and *miR-214* are involved in melanoma growth and metastasis formation by modulating several target genes. We are therefore interested in performing discovery analysis searching for STPs involving these two miRNAs. This represents a typical biological question for which CyTRANSFINDER can provide explorative analysis support.

### STPs involving human miR-146a analysis

Human *miR-146a* is located on the positive strand of chromosome 5. Although it is an intergenic miRNA and it does not lie inside a host protein-coding gene, it is overlapped to a manually-annotated long-intergenic-noncoding RNA (lincRNA), CTC-231O11.1 ([31] and http://www.ensembl.org). *miR-146a* has a crucial role in the immune and inflammatory response, as well as in many human pathologies including muscle disorders, cancer and metastasis [31]. We and others found that *miR-146a* has a dual role during melanoma development and progression, favoring primary tumor growth while inhibiting metastatic dissemination [32]. We are interested in exploring STPs involving *miR-146a* to identify new regulatory paths of interest for the melanoma progression. In order to exploit CyTRANSFINDER for this purpose we need to create a SRL and a DGL file.

Our SRL list contains the *miR-146a* (miRBase identifier hsa-mir-146a) [see Additional file 1 – srl.txt]. We considered instead the set of *miR-146a* conserved target genes according to TargetScan 5.2 algorithm (224 genes) as DGL [see Additional file 1 – dgl.txt].

We performed an analysis using the Indirect s. miRNA STPP using the default setting of the plugin for all filters, and we have been able to identify a set of 312 STPs [see Additional file 1 – Indirect-miRNA-STPP] starting from *miR-146a*. Among all, we got particularly interested in *TFAP2C* (*AP-2 γ*). In our previous studies, we identified the central role of *TFAP2C* in melanoma progression, and we are particularly interested in studying the STPs involving this transcription factor ([33, 34]).

**Fig. 5:**
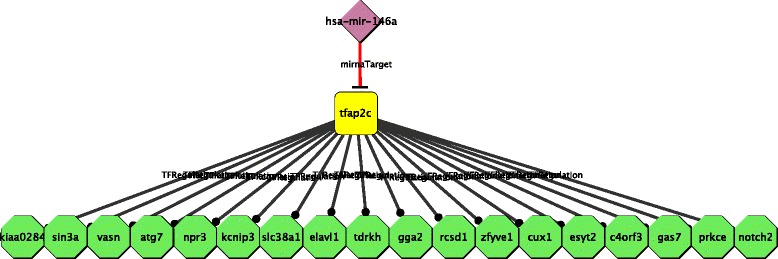
*miR-146a* Indirect s. miRNA STPs involving *TFAP2C*. Subnetwork of the Indirect s. miRNA STPs using human *miR-146a* as source intergenic miRNA, *miR-146a* targets according to TargetScan 5.1 as destination genes and involving *TFAP2C* as a hub transcription factor. Results are computed using miRNA targets confirmed in at least one source database

All the *TFAP2C*-mediated STPs are listed in Table 2 and the related network is reported in Fig. 5. Notably, *miR-146a* relation with *TFAP2C* was completely unknown.

**Table 2:** List of the Indirect s. miRNA STPs obtained with CyTRANSFINDER using human *miR-146a* as the source intergenic miRNA, *miR-146a* targets according to TargetScan 5.1 as destination genes and involving *TFAP2C* as a hub transcription factor

SmiRNA	TF	DG	Reference
hsa-miR-146a	TFAP2C	PRKCE	
hsa-miR-146a	TFAP2C	GAS7	
hsa-miR-146a	TFAP2C	C4ORF3	
hsa-miR-146a	TFAP2C	ESYT2	
hsa-miR-146a	TFAP2C	CUX1	
hsa-miR-146a	TFAP2C	ZFYVE1	
hsa-miR-146a	TFAP2C	RCSD1	
hsa-miR-146a	TFAP2C	ELAVL1	
hsa-miR-146a	TFAP2C	SLC38A1	
hsa-miR-146a	TFAP2C	KCNIP3	
hsa-miR-146a	TFAP2C	ATG7	
hsa-miR-146a	TFAP2C	VASN	
hsa-miR-146a	TFAP2C	SIN3A	
hsa-miR-146a	TFAP2C	KIAA0284	
hsa-miR-146a	TFAP2C	GGA2	[36]
hsa-miR-146a	TFAP2C	NOTCH2	[36]
hsa-miR-146a	TFAP2C	NPR3	[36]
hsa-miR-146a	TFAP2C	TDRKH	[36]

Results are computed using miRNA targets confirmed in at lease one source database. SmiRNA: Source intergenic miRNA; TF: Transcription Factor; DG: Destination Gene; Reference: data available from literature

Given the interest of this result, we performed gene expression analysis via quantitative Real Time Polymerase Chain Reaction (qRT-PCR) to further investigate this relation. To obtain transient *miR-146a* or non-specific control (pre-Cntrl) expression, human melanoma *MA-2* cells (cultured as in [33]) were transfected using HiPerFect (Qiagen) reagent, according to the manufacturer’s instructions. Total RNA was isolated 48h later from using TRIzol®;Reagent (Invitrogen Life Technologies). 1 *μ*g of DNAse-treated RNA (RQ1 RNase-Free DNase, Promega) was retrotranscribed with High-Capacity cDNA Reverse Transcription Kit (Thermo Fisher Scientific,) and qRT-PCRs were carried out using gene-specific primers for TFAP2C mRNA detection (fw: TCCACGACATGCCTCACCA, rv: TCCTTCTGACAGGGGAGGTTCA).

Quantitative normalization was performed on the expression of the *GAPDH* gene (qRT-PCR QuantiTect Primer assay QT01192646, Qiagen). The relative expression levels between samples were calculated using the comparative delta Ct (threshold cycle number) method (2^−*ΔΔCt*^) with a control sample as the reference point [35].

**Fig. 6:**
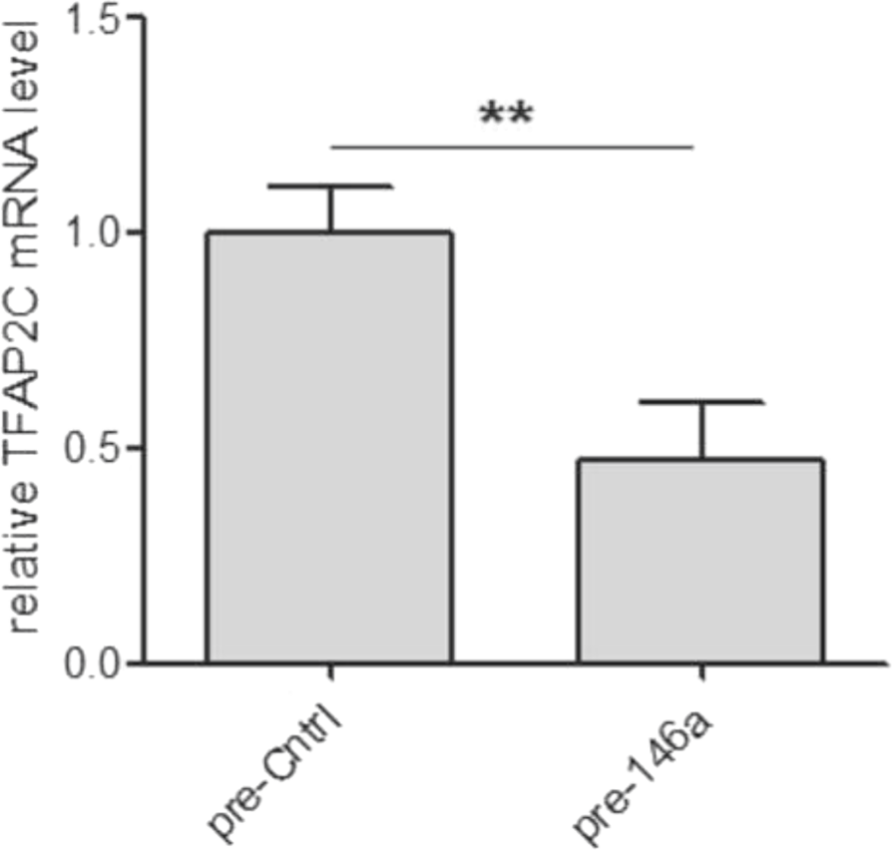
*miR-146a* overexpression leads to reduced *TFAP2C* mRNA levels. Quantitative-Real Time PCR (qRT-PCR) evaluation of *TFAP2C* mRNA was performed in melanoma cells upon *miR-146a* overexpression, compared to controls (pre-146a vs pre-Cntrl). Three independent preparations of melanoma cells RNA were used and results were pooled together. **P <0.01

Data are presented as mean ± s.e.m. (standard error of the mean) and Two tailed Student’s t-test was used for comparison, with **P <0.01 considered to be statistically significant. Based on these experiments we were able to experimentally verify that *miR-146a* is able to downmodulate *TFAP2C* expression upon transient overexpression in human melanoma cells (see Fig. 6).

Furthermore, by searching the literature for TF ↦ gene STPs involving *TFAP2C* and our *DGL*, we found a paper by Woodfield and colleagues where direct regulation by *TFAP2C* on *GGA2*, *NOTCH2*, *NPR3* and *TDRKH* promoter regions was demonstrated by chromatin immunoprecipitation followed by sequencing (ChIP-Seq) analysis [36], as shown in Table 2.

**Fig. 7:**
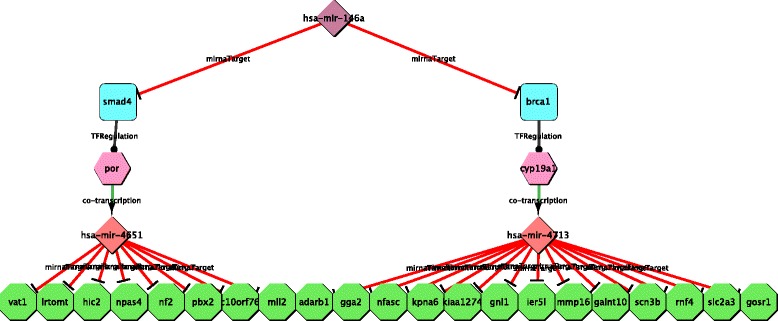
*miR-146a* Double Indirect s. miRNA STPs. Network of the Double Indirect s. miRNA STPs using human *miR-146a* as source intergenic miRNA, *miR-146a* targets according to TargetScan 5.1 as destination genes and miRNA targets confirmed in at least two of the source databases

Next, we also searched for Double Indirect s. miRNA STPP involving *miR-146a* and the selected DGL. Given the complexity of this pattern, we performed the analysis restricting to miRNA targets confirmed in at least two databases out of the four available in TargetHUB. In this case, we obtained a significantly shorter list of records, that is reported in Table 3 and is visually reproduced in Fig. 7.

**Table 3:** List of Double Indirect s. miRNA STPs obtained with CyTRANSFINDER using human *miR-146a* as the source intergenic miRNA, targets according to TargetScan 5.1 as destination genes and miRNA targets confirmed in at least two of the source databases

SmiRNA	References	TF	References	TF target (Gene)	ImiRNA	DG
has-miR-146a	[37, 50–52]	SMAD4	[39]	POR	has-miR-4651	MLL2
has-miR-146a	[37, 50–52]	SMAD4	[39]	POR	has-miR-4651	C10ORF76
has-miR-146a	[37, 50–52]	SMAD4	[39]	POR	has-miR-4651	PBX2
has-miR-146a	[37, 50–52]	SMAD4	[39]	POR	has-miR-4651	NF2
has-miR-146a	[37, 50–52]	SMAD4	[39]	POR	has-miR-4651	NPAS4
has-miR-146a	[37, 50–52]	SMAD4	[39]	POR	has-miR-4651	HIC2
has-miR-146a	[37, 50–52]	SMAD4	[39]	POR	has-miR-4651	LRTOMT
has-miR-146a	[37, 50–52]	SMAD4	[39]	POR	has-miR-4651	VAT1
has-miR-146a	[53]	BRCA1	[40, 54, 55]	CYP19A1	has-miR-4713	GOSR1
has-miR-146a	[53]	BRCA1	[40, 54, 55]	CYP19A1	has-miR-4713	SLC2A3
has-miR-146a	[53]	BRCA1	[40, 54, 55]	CYP19A1	has-miR-4713	RNF4
has-miR-146a	[53]	BRCA1	[40, 54, 55]	CYP19A1	has-miR-4713	SCN3B
has-miR-146a	[53]	BRCA1	[40, 54, 55]	CYP19A1	has-miR-4713	GALNT10
has-miR-146a	[53]	BRCA1	[40, 54, 55]	CYP19A1	has-miR-4713	MMP16
has-miR-146a	[53]	BRCA1	[40, 54, 55]	CYP19A1	has-miR-4713	IER5L
has-miR-146a	[53]	BRCA1	[40, 54, 55]	CYP19A1	has-miR-4713	GNL1
has-miR-146a	[53]	BRCA1	[40, 54, 55]	CYP19A1	has-miR-4713	KIAA1274
has-miR-146a	[53]	BRCA1	[40, 54, 55]	CYP19A1	has-miR-4713	KPNA6
has-miR-146a	[53]	BRCA1	[40, 54, 55]	CYP19A1	has-miR-4713	NFASC
has-miR-146a	[53]	BRCA1	[40, 54, 55]	CYP19A1	has-miR-4713	GGA2
has-miR-146a	[53]	BRCA1	[40, 54, 55]	CYP19A1	has-miR-4713	ADARB1

SmiRNA: Source intergenic miRNA; TF: Transcription Factor; TF target (Gene): Transcription Factor target gene, which is also the host gene for a miRNA; ImiRNA: miRNA located inside the TF target gene; DG: Destination Gene; Reference: data available from literature

Notably, the STPs identified by CyTRANSFINDER seem of biological relevance, since our paths link *miR-146a* to TFs *SMAD4* and *BRCA1* (miRNA ↦ TF regulation), as well-established in literature (Table 3). *SMAD4* is a key transcription factor involved in the *TGF*- *β* mediated response [37], while *BRCA1* is involved in the DNA damage repair and is one of the main mutated genes in familial breast and ovarian cancers [38]. *SMAD4*- and *BRCA1*- regulated genes (TF target (gene)) that we obtained, *POR* and *CYP19A1* respectively, are again well-established in literature (Table 3), and their involvement downstream of *miR-146a* could be very interesting, since they both have a role in hormones production and cancer. *POR* gene codifies for cytochrome *P450* oxidoreductase enzyme, which catalyzes the biosynthesis of steroid hormones and metabolize drugs [39], while *CYP19A1* codes for the aromatase enzyme that converts androgens into estrogens, and which dysregulation may affect estrogen production in breast cancer cases [40]. *POR* and *CYP19A1* genes host one microRNA each, *miR-4651* and *miR-4713*, respectively (gene ↦ miRNA regulation; they are indicated as Intragenic microRNA). *miR-4651* and *miR-4713* target genes predicted by at least 2 algorithms are listed in Table 3 (DG), but, unfortunately, since they are recently discovered miRNAs, none of their target genes has been experimentally validated yet.

### STPs involving human miR-214 analysis

Human *miR-214* gene is located in the chromosomal region 1q24.3, in intron 14 of the Dynamin-3 gene (*DNM3*) inside an almost 8 kb-long noncoding RNA, named *DNM3os*. This transcript contains the sequences for *miR-214* and *miR-199a-2*, two clustered miRs that are approximately 6 kb apart. *miR-214* is deregulated in a variety of human tumors including melanoma, breast, ovarian, gastric, and hepatocellular carcinomas as reviewed in [41]. In melanoma, we demonstrated that *miR-214* has essential roles in regulating invasiveness, extravasation and metastasis formation [33, 34]. In particular, we identified a signature of 73 genes whose expression was driven by miR-214 [33].

In order to identify new molecular pathways underlying *miR-214*-mediated regulation of these genes we took advantage of CyTRANSFINDER. Differently from the previous case, to show the use of the software on a STPP starting from a gene, we used *DNM3*, the host gene of *miR-214*, as SRL [see Additional file 2 – srl.txt] and the *miR-214*-dependent signature mentioned above as the DGL [see Additional file 2 – dgl.txt]. We searched for Double miRNA Indirect STPs identifying 312 STPs involving different transcription factors (TFs), miRNA host genes (TF target gene) targeted by these TFs and cognate intragenic miRNAs (ImiRNAs) as nodes [see Additional file 2 – Double-indirect-miRNA-STPP.xlsx].

**Fig. 8:**
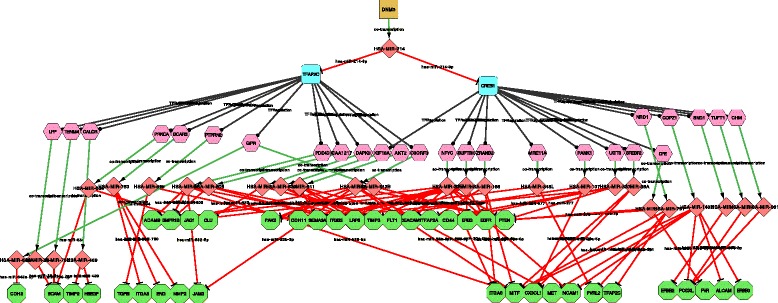
*miR-214* Double Indirect miRNA STPs. Sub-Network of a selection of 101 interesting Double Indirect miRNA STPs using human DNM3 as source gene, a signature of 73 genes published in [33] as destination genes, and involving *TFAP2C* and *CREB1* transcription factors. Results are computed using miRNA targets confirmed in at least two databases

Interestingly enough, the majority of the STPPs were controlled by two of the most relevant TFs for melanoma biology, the transcription factor *AP-2* gamma *TFAP2C* (*AP-2 γ*) [33] and the *cAMP* responsive element binding protein 1, *CREB1* [42]. We focused our attention on the STPs driven by these two TFs and we selected a subgroup (101) of STPPs, containing well-described intragenic miRNAs (Intragenic microRNAs) as nodes (Table 4 and Fig. 8).

**Table 4:** List of a selection (101) of the Double Indirect miRNA STPPs obtained with CyTRANSFINDER using the host gene of *miR-214*, *DNM3*, as the source gene, a signature of 73 genes published in [33] as destination genes, and involving *TFAP2C* and *CREB1* transcription factors

SmiRNA	References	TF	TF target (Gene)	ImiRNA	References	DG
hsa-mir-214	[33]	TFAP2C	C9ORF3	HSA-MIR-27B-3p		LRP6
hsa-mir-214	[33]	TFAP2C	C9ORF3	HSA-MIR-27B-3p	[56, 57]	MET
hsa-mir-214	[33]	TFAP2C	C9ORF3	HSA-MIR-27B-3p		NCAM1
hsa-mir-214	[33]	TFAP2C	C9ORF3	HSA-MIR-27B-3p	[57–59]	ARHGAP12
hsa-mir-214	[33]	TFAP2C	C9ORF3	HSA-MIR-27B-3p		HBEGF
hsa-mir-214	[33]	TFAP2C	C9ORF3	HSA-MIR-27B-3p		EGFR
hsa-mir-214	[33]	TFAP2C	C9ORF3	HSA-MIR-27B-3p	[56]	MITF
hsa-mir-214	[33]	TFAP2C	C9ORF3	HSA-MIR-27B-3p	[60]	CDH11
hsa-mir-214	[33]	TFAP2C	C9ORF3	HSA-MIR-23B		MITF
hsa-mir-214	[33]	TFAP2C	C9ORF3	HSA-MIR-23B	[61, 62]	PAK2
hsa-mir-214	[33]	TFAP2C	C9ORF3	HSA-MIR-23B	[56, 62, 63]	PTEN
hsa-mir-214	[33]	TFAP2C	C9ORF3	HSA-MIR-23B		BMPR1B
hsa-mir-214	[33]	TFAP2C	C9ORF3	HSA-MIR-23B		JAM3
hsa-mir-214	[33]	TFAP2C	C9ORF3	HSA-MIR-23B	[56, 62]	MET
hsa-mir-214	[33]	TFAP2C	BCAR3	HSA-MIR-760		MMP2
hsa-mir-214	[33]	TFAP2C	BCAR3	HSA-MIR-760		ITGA3
hsa-mir-214	[33]	TFAP2C	BCAR3	HSA-MIR-760		ENG
hsa-mir-214	[33]	TFAP2C	CALCR	HSA-MIR-489		HBEGF
hsa-mir-214	[33]	TFAP2C	CALCR	HSA-MIR-489		TIMP2
hsa-mir-214	[33]	TFAP2C	PTPRN2	HSA-MIR-595		TGFBI
hsa-mir-214	[33]	TFAP2C	PTPRN2	HSA-MIR-595		CDH11
hsa-mir-214	[33]	TFAP2C	KIAA1217	HSA-MIR-603		TIMP3
hsa-mir-214	[33]	TFAP2C	KIAA1217	HSA-MIR-603		BMPR1B
hsa-mir-214	[33]	TFAP2C	KIAA1217	HSA-MIR-603		ITGB3
hsa-mir-214	[33]	TFAP2C	PRKCA	HSA-MIR-634		BCAM
hsa-mir-214	[33]	TFAP2C	PRKCA	HSA-MIR-634		JAG1
hsa-mir-214	[33]	TFAP2C	PDE4D	HSA-MIR-582		LRP6
hsa-mir-214	[33]	TFAP2C	PDE4D	HSA-MIR-582		MITF
hsa-mir-214	[33]	TFAP2C	PDE4D	HSA-MIR-582		BMPR1B
hsa-mir-214	[33]	TFAP2C	PDE4D	HSA-MIR-582		JAM3
hsa-mir-214	[33]	TFAP2C	PDE4D	HSA-MIR-582		ADAM9
hsa-mir-214	[33]	TFAP2C	LPP	HSA-MIR-28		BCAM
hsa-mir-214	[33]	TFAP2C	GIPR	HSA-MIR-642A		CDH2
hsa-mir-214	[33]	TFAP2C	GIPR	HSA-MIR-642B		PTEN
hsa-mir-214	[33]	TFAP2C	GIPR	HSA-MIR-642B		PAK2
hsa-mir-214	[33]	TFAP2C	TENM4	HSA-MIR-708		BCAM
hsa-mir-214	[33]	TFAP2C	AKT2	HSA-MIR-641		TIMP3
hsa-mir-214	[33]	TFAP2C	AKT2	HSA-MIR-641		TFAP2A
hsa-mir-214	[33]	TFAP2C	AKT2	HSA-MIR-641		LRP6
hsa-mir-214	[33]	TFAP2C	AKT2	HSA-MIR-641		SEMA3A
hsa-mir-214	[33]	TFAP2C	DAPK3	HSA-MIR-637		FLT1
hsa-mir-214	[33]	TFAP2C	DAPK3	HSA-MIR-637		CLU
hsa-mir-214	[33]	TFAP2C	ZRANB2	HSA-MIR-186		ITGA6
hsa-mir-214		CREB1	ZRANB2	HSA-MIR-186-5p	[59]	TFAP2A
hsa-mir-214		CREB1	ZRANB2	HSA-MIR-186		JAG1
hsa-mir-214		CREB1	ZRANB2	HSA-MIR-186		MITF
hsa-mir-214		CREB1	SUPT3H	HSA-MIR-586		FLT1
hsa-mir-214		CREB1	SUPT3H	HSA-MIR-586		EREG
hsa-mir-214		CREB1	SUPT3H	HSA-MIR-586		EGFR
hsa-mir-214		CREB1	SUPT3H	HSA-MIR-586		SEMA3A
hsa-mir-214		CREB1	KIF18A	HSA-MIR-610		TIMP3
hsa-mir-214		CREB1	KIF18A	HSA-MIR-610		LRP6
hsa-mir-214		CREB1	UGT8	HSA-MIR-577		CD44
hsa-mir-214		CREB1	UGT8	HSA-MIR-577		TFAP2A
hsa-mir-214		CREB1	UGT8	HSA-MIR-577		PTEN
hsa-mir-214		CREB1	TUFT1	HSA-MIR-554		PODXL
hsa-mir-214		CREB1	SREBF2	HSA-MIR-33A		CDH11
hsa-mir-214		CREB1	SREBF2	HSA-MIR-33A		MITF
hsa-mir-214		CREB1	SREBF2	HSA-MIR-33A		CX3CL1
hsa-mir-214		CREB1	PANK1	HSA-MIR-107		CX3CL1
hsa-mir-214		CREB1	PANK1	HSA-MIR-107		LRP6
hsa-mir-214		CREB1	NRD1	HSA-MIR-761		TFAP2C
hsa-mir-214		CREB1	NRD1	HSA-MIR-761		PVRL2
hsa-mir-214		CREB1	NRD1	HSA-MIR-761		MITF
hsa-mir-214		CREB1	NFYC	HSA-MIR-30E		LRP6
hsa-mir-214		CREB1	NFYC	HSA-MIR-30E		CEACAM1
hsa-mir-214		CREB1	NFYC	HSA-MIR-30E		ITGA6
hsa-mir-214		CREB1	NFYC	HSA-MIR-30E		PTEN
hsa-mir-214		CREB1	NFYC	HSA-MIR-30E		ADAM9
hsa-mir-214		CREB1	NFYC	HSA-MIR-30E		SEMA3A
hsa-mir-214		CREB1	NFYC	HSA-MIR-30E		ITGB3
hsa-mir-214		CREB1	NFYC	HSA-MIR-30E		NCAM1
hsa-mir-214		CREB1	NFYC	HSA-MIR-30E-3p	[60]	TIMP3
hsa-mir-214		CREB1	MRE11A	HSA-MIR-548L		PVRL2
hsa-mir-214		CREB1	MRE11A	HSA-MIR-548L		PAK2
hsa-mir-214		CREB1	SND1	HSA-MIR-593		ERBB2
hsa-mir-214		CREB1	TMEM245	HSA-MIR-32-5p	[56, 58, 59]	ITGA6
hsa-mir-214		CREB1	TMEM245	HSA-MIR-32-5p		PTEN
hsa-mir-214		CREB1	TMEM245	HSA-MIR-32-5p	[64]	SEMA3A
hsa-mir-214		CREB1	TMEM245	HSA-MIR-32-5p	[65]	ITGAV
hsa-mir-214		CREB1	TMEM245	HSA-MIR-32-5p		MITF
hsa-mir-214		CREB1	COPZ1	HSA-MIR-148B	[66]	ERBB3
hsa-mir-214		CREB1	COPZ1	HSA-MIR-148B	[34]	ALCAM
hsa-mir-214		CREB1	COPZ1	HSA-MIR-148B		MET
hsa-mir-214		CREB1	COPZ1	HSA-MIR-148B		NCAM1
hsa-mir-214		CREB1	COPZ1	HSA-MIR-148B		PODXL
hsa-mir-214		CREB1	COPZ1	HSA-MIR-148B	[63]	PTEN
hsa-mir-214		CREB1	COPZ1	HSA-MIR-148B		MITF
hsa-mir-214		CREB1	CPE	HSA-MIR-578		PVR
hsa-mir-214		CREB1	CPE	HSA-MIR-578		PTEN
hsa-mir-214		CREB1	CHM	HSA-MIR-361		PODXL
hsa-mir-214		CREB1	SMC4	HSA-MIR-15B-5p	[64]	SEMA3A
hsa-mir-214		CREB1	SMC4	HSA-MIR-15B-5p	[63, 67]	APP
hsa-mir-214		CREB1	SMC4	HSA-MIR-15B-5p	[58]	PAK2
hsa-mir-214		CREB1	SMC4	HSA-MIR-15B-5p		PODXL
hsa-mir-214		CREB1	SMC4	HSA-MIR-15B-5p		PVRL2
hsa-mir-214		CREB1	SMC4	HSA-MIR-15B-5p	[65]	TFAP2A
hsa-mir-214		CREB1	SMC4	HSA-MIR-15B-5p	[63]	ARHGAP12
hsa-mir-214		CREB1	SMC4	HSA-MIR-15B-5p		KDR
hsa-mir-214		CREB1	SMC4	HSA-MIR-15B-5p		CX3CL1
hsa-mir-214		CREB1	SMC4	HSA-MIR-15B-5p		LRP6

Results are computed using miRNA targets confirmed in at least two databases. smiRNA name: source intragenic microRNA; TF: Transcription Factor; TF target (Gene): Transcription Factor target gene, which is also the host gene for a miRNA; ImiRNA: miRNA located inside the TF target gene; DG: Destination Gene, list of targets of the intragenic miRNAs predicted by at least two algorithms; Reference: data available from literature

Searching the literature for potential validations of these STPPs, we were able to find partial validations. The connection between *miR-214* and *TFAP2C* was clearly demonstrated in our previous work [33], where we showed the direct targeting of *miR-214* on *TFAP2C* 3 ′-UTR; while no data linking *miR-214* and CREB1 were found. No connections were observed for either *TFAP2C* or *CREB1* and the host genes of intragenic miRNAs (TF target) present in the STPs, except for the phospholipid-dependent protein-serine/threonine kinase *PRKCA* gene. *PRKCA* plays a major role in intracellular signaling pathways associated with transformation and tumor progression and its expression was shown to be under the control of *TFAP2* transcription factor family [43].

Looking for potential targeting of the analyzed intragenic miRNAs (Intragenic microRNA) on genes of the DGL we found numerous experimental validations in the literature. In particular, we were interested in STPPs driven by *CREB1* since potential *miR-214* ↦*CREB1* connections could open up new lines of research in understanding *miR-214*-driven metastatization. Among *CREB1*-controlled STPs we found *SREBP2* (TF target) and *miR-33a* (Intragenic microRNA) that are known to be co-regulated [44] and we previously demonstrated to be downregulated by *miR-214* [45]. Very recently, Zhou and colleagues demonstrated *miR-33a* tumor suppressive role in melanoma, thus suggesting a potential additional effect of *miR-214* in promoting melanoma malignancy via the downregulation of another miRNA, miR-33a [46]. We demonstrated the ability of *miR-214* to promote melanoma progression by downregulation of *miR-148b* at least partially via *TFAP2C* regulation, thus leading to *miR-148b* targets derepression, such as *ALCAM* [34]. Interestingly enough, we were able to find *miR-148b* and *ALCAM* in one of the STPs, but surprisingly from CyTRANSFINDER analysis *CREB1*, and not *TFAP2C*, resulted to be the master regulator of the pathway. These new data are very interesting for us and we would like to investigate this potential pathway more in detail. In fact, it has been demonstrated that *CREB1* is able to regulate *TFAP2A* expression in melanoma [47], so we could hypothesize a double control of *miR-214* on *TFAP2C*, direct, via targeting, and, indirect, via *CREB1*, thus leading to a strong promotion of melanoma progression. Finally, another STP interestingly linked *miR-214* to *miR-15b-5p*. In particular, 5 (*SEMA3A*, *APP*, *PAK2*, *TFAP2A* and *ARHGAP12*) out of 10 DGL genes resulted to be validated targets of this miRNA and moreover, *miR-15b* was shown to be involved in tumor cell proliferation and apoptosis in malignant melanoma [48].

## Conclusions

Here we presented a new plugin for Cytoscape, CyTRANSFINDER that provides support to discover three-component signal transduction pathways with the presence of miRNAs, TFs and genes. Differently from other tools, the plugin is specifically designed to perform exploratory analysis and to identify new biological circuits to be tested in laboratory. Therefore, it only relies on aggregation of complex repositories without requiring any expression data.

To show the capabilities of this plugin we applied it to a real use case involving the study of two miRNAs that are particularly relevant in human melanoma progression. Taken together, our analyses on the STPs generated by CyTRANSFINDER unravelled many relevant potential pathways regulated by *miR-146a* and *miR-214* in human physiology and pathology; some of these are validated in literature, while others were validated by us.

This should give a clear view of the potential this tool has to support biologists in discovering novel signal transduction pathways regulated by miRNAs and transcription factors.

A detailed tutorial containing a step-by-step guide covering all CyTRANSFINDER features is available at http://apps.cytoscape.org/apps/cytransfinder.

We are currently at the first release of this tool. One of the critical aspects of this implementation is the huge amount of data the plugin needs to retrieve from the Internet and to process internally. This in turns requires processing time that ranges from a few minutes for very simple lists of genes to several hours for complex lists of genes such as the one used in the proposed case studies. Future releases will address this specific aspect by introducing several caching mechanism enabling to reduce the network traffic and to significantly increase the computation speed.

Moreover, we are also interested in inserting new features to make the generated networks as specific as possible. Recently, we discovered mimiRNA, a database of miRNA expression profiles data across different tissues and cell lines [49]. mimiRNA incorporates a sample classification algorithm that groups identical miRNA or mRNA experiments from separate sources and provides reliable expression profiles of miRNA in different tissues and cell lines. We plan to integrate mimiRNA into CyTRANSFINDER as an additional filter enabling to select only intra/intergenic miRNAs that are expressed in a set of tissues or cell lines of interest for the user, thus reducing the size of the generated networks.

Finally, we are evaluating the possibility of defining a scoring approach to help users to select STPs based on different search criteria (e.g., involvement of the identified regulator in a disease based on literature, strength of the single identified interactions based on scores available in other external databases, etc.).

## Ethics

Our study does not involve humans, human data or animals. For this reason no ethical approvals are required.

## Availability and requirements

- **Project name**: CyTRANSFINDER
- **Project home page**: http://apps.cytoscape.org/apps/cytransfinder
- **Operating system(s)**: Platform independent
- **Programming language**: Java
- **Other requirements**: Java 8 or higher, Cytoscape 3.3 (latest version tasted is Cytoscape 3.3)
- **License**: Creative Commons License (http://creativecommons.org/licenses/by-nc-sa/4.0/)
- **Any restrictions to use by non-academics**: Only those imposed already by the license

## Additional files

Additional file 1 ∙ srl.txt: the file containing the SRL. ∙ dgl.txt: the file containing the DGL. ∙ Indirect-miRNA-STPP.xlsx: an excel file containing the full list of 312 Indirect miRNA STPPs identified by CyTRANSFINDER using human *miR-146a* as the source intergenic miRNA and *miR-146a* 223 target genes according to TargetScan 5.2 as DGL. Results are computed using miRNA targets confirmed in at lease one source database. SmiRNA: source intragenic miRNA; TF: Transcription Factor; DG: Destination Gene. (ZIP 201 kb)

Additional file 2 ∙ srl.txt: the file containing the SRL. ∙ dgl.txt: the file containing the DGL. ∙ Indirect-miRNA-STPP.xlsx: an excel file containing the full list of 312 Indirect miRNA STPPs identified by CyTRANSFINDER using human *miR-146a* as the source intergenic miRNA and *miR-146a* 223 target genes according to TargetScan 5.2 as DGL. Results are computed using miRNA targets confirmed in at lease one source database. SmiRNA: source intragenic miRNA; TF: Transcription Factor; DG: Destination Gene. an excel file containing the full list of 292 Double Indirect miRNA STPPs identified by TransFINDER using *DNM3* as the Source Gene, the cognate human intragenic miR-214 as source intragenic miRNA (SmiRNA) and a previously described signature of 73 genes whose expression was driven by miR-214 [33] as destination genes (DG). TF: Transcription Factor; TF target (Gene): Transcription Factor target gene, which is also the host gene for a miRNA; intragenic miRNA: miRNA located inside the TF target gene; DG: Destination Gene, list of targets of the intragenic miRNAs predicted by at least two algorithms TS: Transcription Factor. (ZIP 192 kb)
